# Genomic Signatures of Recent Adaptation in a Wild Bumblebee

**DOI:** 10.1093/molbev/msab366

**Published:** 2022-02-03

**Authors:** Thomas J Colgan, Andres N Arce, Richard J Gill, Ana Ramos Rodrigues, Abdoulie Kanteh, Elizabeth J Duncan, Li Li, Lars Chittka, Yannick Wurm

**Affiliations:** 1 School of Biological and Behavioural Sciences, Queen Mary University of London, London, United Kingdom; 2 Department of Life Sciences, Imperial College London, Silwood Park, Ascot, United Kingdom; 3 School of Biology, Faculty of Biological Sciences, University of Leeds, Leeds, United Kingdom; 4 Alan Turing Institute, London, United Kingdom

**Keywords:** ecological genomics, population genomics, selective sweeps, bees, horizontal gene transfer, genetic health

## Abstract

Environmental changes threaten insect pollinators, creating risks for agriculture and ecosystem stability. Despite their importance, we know little about how wild insects respond to environmental pressures. To understand the genomic bases of adaptation in an ecologically important pollinator, we analyzed genomes of *Bombus terrestris* bumblebees collected across Great Britain. We reveal extensive genetic diversity within this population, and strong signatures of recent adaptation throughout the genome affecting key processes including neurobiology and wing development. We also discover unusual features of the genome, including a region containing 53 genes that lacks genetic diversity in many bee species, and a horizontal gene transfer from a *Wolbachia* bacteria. Overall, the genetic diversity we observe and how it is distributed throughout the genome and the population should support the resilience of this important pollinator species to ongoing and future selective pressures. Applying our approach to more species should help understand how they can differ in their adaptive potential, and to develop conservation strategies for those most at risk.

Behavioral experiments and analyses of observation records have shown that pesticide use, habitat fragmentation, emerging diseases, and climatic change threaten insect pollinators including bees (Brown and Paxton 2009; Vanbergen 2013; Ollerton et al. 2014; Goulson et al. 2015). Despite the resulting risks for agricultural yields and for ecosystem stability, we know little about how wild insects may adapt to such environmental pressures. Similarly, we understand relatively little about how genomes are shaped by selection in the wild (Harrisson et al. 2014). If a species has adapted in response to a detrimental environmental pressure, then we should see changes in the alleles of genes involved (Hohenlohe et al. 2010; Ellegren 2014) (fig. 1*A*). Analyzing genomes of many individuals can identify such changes and reveal the constraints and adaptive potential of species (Chen et al. 2018; van Klink et al. 2020). The resulting knowledge should support conservation efforts and practices (Supple and Shapiro 2018).

**Fig. 1:**
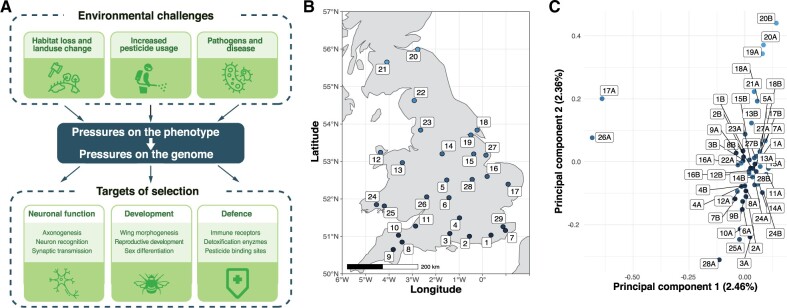
Environmental pressures affecting insect pollinators and population structure of wild-caught British *B. terrestris*. (*A*) Overview of key environmental selective pressures on wild bumblebee populations, and some of the biological pathways and processes expected to be under selection in response. (*B*) Twenty-eight collection sites across Great Britain, colored according to latitude. (*C*) Population structure of 46 males according to the first two principal components (PC1 and PC2). Each point refers to one male, with up to two males (A and B) per site, colored according to collection site latitude.

The annually reproductive bumblebee *Bombus terrestris* is ideal for understanding how coping with recent rates of environmental change is possible because, unlike many other pollinators, it has shown little evidence of population decline (Ollerton et al. 2014). Furthermore, because male bumblebees are haploid, their genome sequences are unambiguous and intrinsically phased, providing more analytical power than the diploid genomes of female bumblebees and of many other insects. To understand which bumblebee genes and molecular processes underlie responses to recent selective pressures, we sequenced the genomes of male *B. terrestris* bumblebees from across Great Britain. We subsequently identified and characterized the genomic regions showing the strongest signs of recent adaptive evolution in this population. Our characterization of the amount and distribution of genetic diversity throughout the bumblebee genome provides measures of the genetic health of this species and highlights genes and processes through which it has recently adapted.

## Results

### Weak Population Substructure among *B. terrestris* in Mainland Britain

We collected 46 unrelated male *B. terrestris* from across Great Britain and sequenced their genomes (411-fold total coverage; fig. 1*B* and supplementary table S1, Supplementary Material online). We found 1,227,312 single-nucleotide polymorphisms (SNPs), with an average nucleotide diversity π of 1.51 × 10^−3^.

To understand whether population structure constrains adaptation in this species, we performed identity-by-state (IBS) and co-ancestry-based analyses. These analyses indicate that our data set represents one population (fig. 1C and supplementary figs. S1 and S2, Supplementary Material online). Similarly, although the second principal component correlates with latitude (Pearson’s *r* = 0.8, fig. 1C and supplementary fig. S3, Supplementary Material online), individual principal components explained at most 2.46% of genetic variation. In line with the relative absence of barriers to gene flow in Great Britain, there is sufficient gene flow for these bees to be considered as one panmictic population, implying that new alleles have the potential to readily spread. The weak substructure of British *B. terrestris* is supported by studies using fewer markers (Schmid-Hempel et al. 2007; Lye et al. 2011; Moreira et al. 2015). Our result also indicates that no subset of our samples has the type of large-scale differentiation that could be expected from a cryptic subspecies.

### Selection Is Fine-Tuning Functional Regions throughout the Bumblebee Genome

**Fig. 2:**
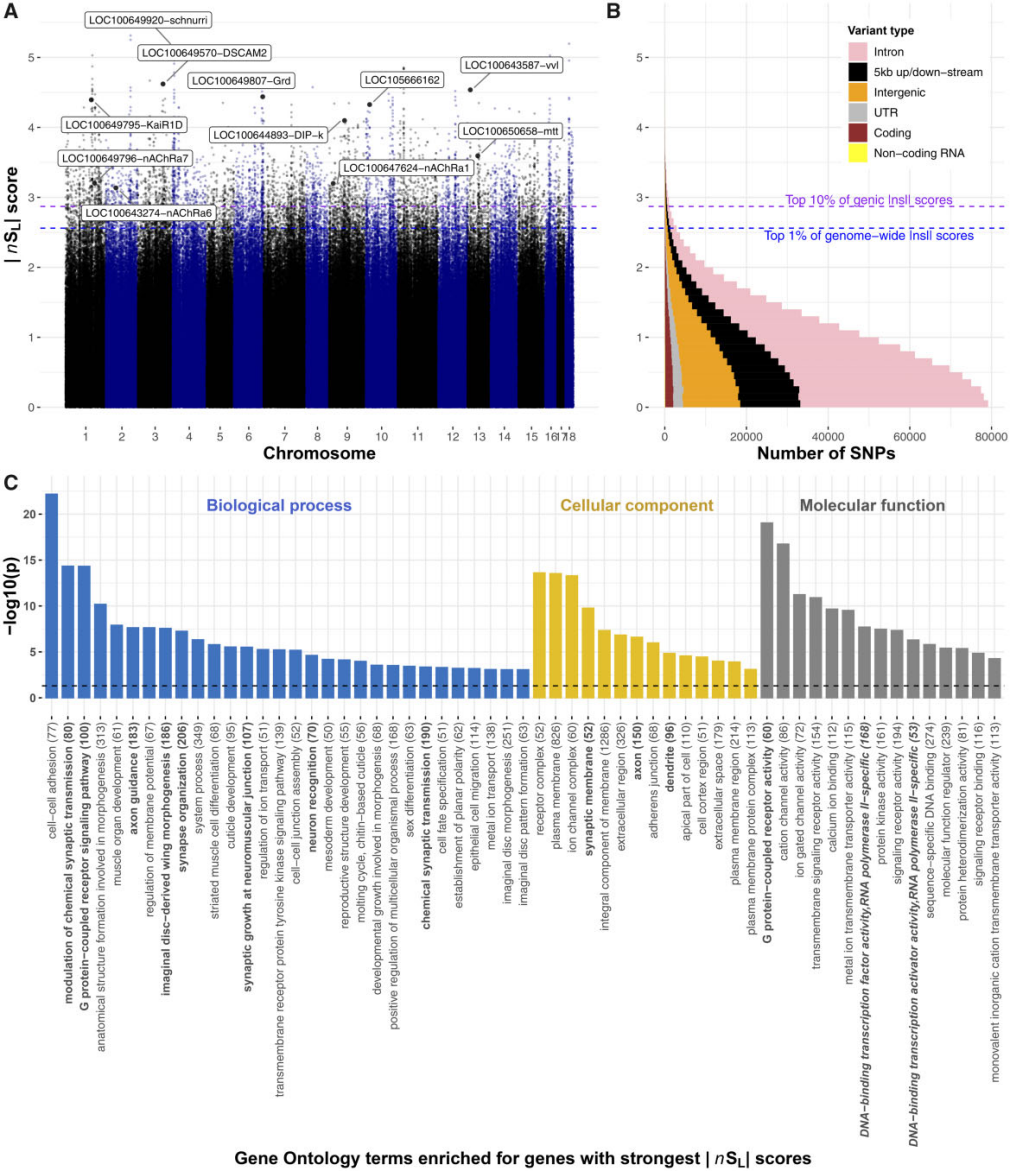
Genome-wide signatures of selective sweeps in British *B. terrestris* bumblebees. (*A*) |*nS*_L_| measures of selection for all SNPs in the bumblebee genome. Each dot represents one SNP; labeled dots represent the SNP with highest |*nS*_L_| score for genes of interest, including transcription factors, insecticide susceptibility genes, and a *Wolbachia*-like gene, with high |*nS*_L_| scores. Labels indicate Flybase gene symbols when clear *Drosophila* orthology exists, otherwise, the NCBI gene symbol is provided. Blue and purple horizontal dashed lines, respectively, indicate the 1st percentile of overall |*nS*_L_| scores and 10th percentile of genic |*nS*_L_| scores. (*B*) Distributions of |*nS*_L_| scores show that most SNPs are in genic regions, and that most |*nS*_L_| scores are consistent with neutral or purifying rather than directional evolution as 96% of SNPs have |*nS*_L_| < 2. (*C*) Diverse Gene Ontology terms are enriched in genes with high |*nS*_L_| scores (−log10 transformed Bonferroni-adjusted *P* values). Terms associated with roles in neurology and transcription factor activity are, respectively, highlighted in bold and bold italics. The total number of annotated genes for each term is in parentheses.

We used two approaches to identify signatures of recent selection in the genome. First, we identified large “hard” sweep regions, where selection on an allele can lead to haplotype fixation (Berry et al. 1991). For this, we identified genomic segments longer than 100,000 nucleotides with significantly lower nucleotide diversity than the rest of the genome (*z*-score < −2*σ*). We found 90 such segments. Our second approach detected more localized signatures of selection, and “soft” sweeps, where two or more haplotypes are at high frequency. This can occur, for example, when strong selection on a new mutation occurs after a first allele reaches fixation, or when selection favors different alleles in different habitats (Hermisson and Pennings 2005). For this, we determined for each SNP the metric |*nS*_L_| (“number of segregating sites by length”), a measure of haplotype homozygosity that is robust to variation in recombination and mutation rates; an |*nS*_L_| score greater than 2 is considered evidence of recent selection (Ferrer-Admetlla et al. 2014; Szpiech and Hernandez 2014). The 10,132 SNPs with the highest 1% of |*nS*_L_| scores have particularly strong signatures of recent selection (|*nS*_L_|>2.56, i.e., ≳3 standard deviations from the mean; fig. 2*A* and supplementary table S2, Supplementary Material online) and are typically within 300 nucleotides of SNPs with low |*nS*_L_| scores. This indicates that recombination rapidly breaks down haplotypes in this species and that selection has acted in fine-tuned manners. The SNPs with high |*nS*_L_| scores are mostly in genic regions (79%; *P *<* *10^−15^) and, within these regions, are similarly represented in coding and noncoding sequence (*P *>* *0.05), indicating that selection recently acted on protein function and on gene regulation.

To understand which types of biological functions were under the strongest recent selection pressures, we inspected annotations of genes with the strongest |*nS*_L_| scores and performed rank-based analyses of Gene Ontology and InterPro descriptions of all bumblebee genes (Bonferroni adjusted *P *<* *0.05; fig. 2*C* and supplementary fig. S4 and tables S3 and S4, Supplementary Material online). The overviews of loci under recent selection, and the biological and molecular processes they affect, represent valuable resources for future phenotypic work on bees and on adaptation in natural insect populations (fig. 2*C* and supplementary fig. S4 and tables S3 and S4, Supplementary Material online). Below, we highlight five particularly striking patterns regarding genes and regions with the strongest signatures of selective sweeps.

#### Strong Selection on Transcription Factors

Genes related to transcriptional regulation were overrepresented among genes under selection (fig. 2*C* and supplementary fig. S4 and tables S3 and S4, Supplementary Material online). In particular, the gene with the strongest evidence of recent selection is the *B. terrestris* ortholog to the *schnurri* gene (|*nS*_L_|=5.14). In *Drosophila*, this transcription factor regulates embryonic patterning and wing patterning through the Decapentaplegic pathway (Torres-Vazquez et al. 2000). Another transcription factor, the ortholog to the *ventral veins lacking* gene (*vvl*), has the ninth highest |*nS*_L_| score (|*nS*_L_|=4.54). In *Drosophila*, *vvl* is involved in steroid biosynthesis and embryonic brain development (Meier et al. 2006; Danielsen et al. 2014), and intriguingly also interacts with the Decapentaplegic pathway to affect wing imaginal disc development (Certel et al. 2000) and vein patterning (de Celis et al. 1995). These results, together with “wing morphogenesis” being the eighth most overrepresented Gene Ontology description among genes under selection, suggest that there was strong recent selection on wing structure. Such selection could be linked to recent changes to foraging or flight patterns (Memmott et al. 2007; Miller-Struttmann et al. 2015), because climatic changes modified the physical constraints of flying (Corbet et al. 1993), or perhaps in response to pathogens, such as the deformed wing virus, which can cause extensive wing abnormalities in infected individuals (Genersch et al. 2006).

#### Strong Selection Acting on Genes Involved in Bumblebee Neurobiology

Neurological genes were overrepresented among genes with the highest |*nS*_L_| scores (fig. 2*C*). In line with this, 4 of the 30 genes with the highest |*nS*_L_| scores have potential roles in neurotransmission (*gamma-aminobutyric acid receptor alpha-like*; |*nS*_L_|=4.44, *glutamate receptor ionotropic kainate 2*; |*nS*_L_|=4.39), axon guidance (*Down Syndrome cell adhesion molecule 2*; |*nS*_L_|=4.62), and memory formation (*neurotrimin*; |*nS*_L_|=4.1). Furthermore, selection on G protein-coupled receptor signaling in *B. terrestris* mirrors previous analyses on honeybees (Harpur et al. 2014; Wallberg et al. 2014; Avalos et al. 2017). These receptor targets of hormones, pheromones, and neurotransmitters are thus long-term targets of selection in social bees, potentially for roles responding to social or to environmental cues (Hauser et al. 2006). Selection on neurological genes could, for example, be linked to the need to improve complex cognitive and social behaviors of bumblebees (Loukola et al. 2017), for remembering increasingly complex foraging routes due to patchier habitats, or for neurological changes because of exposure to neurotoxins.

#### Positive Selection on a Gene Horizontally Transferred from *Wolbachia*

We used similarity searches to understand the potential functions of uncharacterized genes among the genes with the twenty highest |*nS*_L_| scores. This showed that the gene with the 16th-strongest signature of selection (LOC105666162; |*nS*_L_|=4.33; supplementary table S2 and fig. S5, Supplementary Material online) was horizontally transferred to *Bombus* from *Wolbachia*, a genus of bacterial endosymbionts that infect many invertebrates. Indeed, LOC105666162 has strong similarity to a gene in *Wolbachia* (BlastP *e*-values < 10^−16^) but not to most other insects (supplementary fig. S6 and table S5, Supplementary Material online). The data overwhelmingly suggest that this gene is integrated into the *Bombus* genome and not an artifact of potential contamination (supplementary information, Supplementary Material online). Indeed, the same two orthologs flank LOC105666162 in *B. terrestris* and in *B. impatiens*, and this gene is present in genomic sequences of 14 other *Bombus* species (Sadd et al. 2015; Lin et al. 2019), suggesting that horizontal transfer occurred approximately 40 Ma (Peters et al. 2017). Additionally, sequencing depth across LOC105666162 is similar to the rest of the genome (paired *t*-test, *t*_df=95_ = −0.73, *P *=* *0.47). Crucially, we find no other *Wolbachia*-related sequences in our samples, consistent with the absence of evidence that *Wolbachia* could infect *Bombus.* So, what might LOC105666162 do? Unfortunately, functional work on this gene and its *Wolbachia* homolog WD0147 is lacking, but we do have some clues. Horizontally transferred genes are often inactive, yet LOC105666162 is expressed in germlines and other tissues of both sexes and castes (Harrison et al. 2015; Lewis et al. 2018; Colgan et al. 2019), consistent with it being functional (supplementary fig. S5 and table S6, Supplementary Material online). The *Wolbachia* homolog is expressed in infected *Drosophila* gonads (Papafotiou et al. 2011); based on its amino acid sequence and expression profiles, this gene is a strong candidate for driving mechanisms of cytoplasmic incompatibility (Metcalf et al. 2014)*.* One could speculate that LOC105666162 contributes to the lack of *Wolbachia* in bumblebees.

#### An Evolutionary Conserved Region of Extremely Low Genetic Diversity

**Fig. 3:**
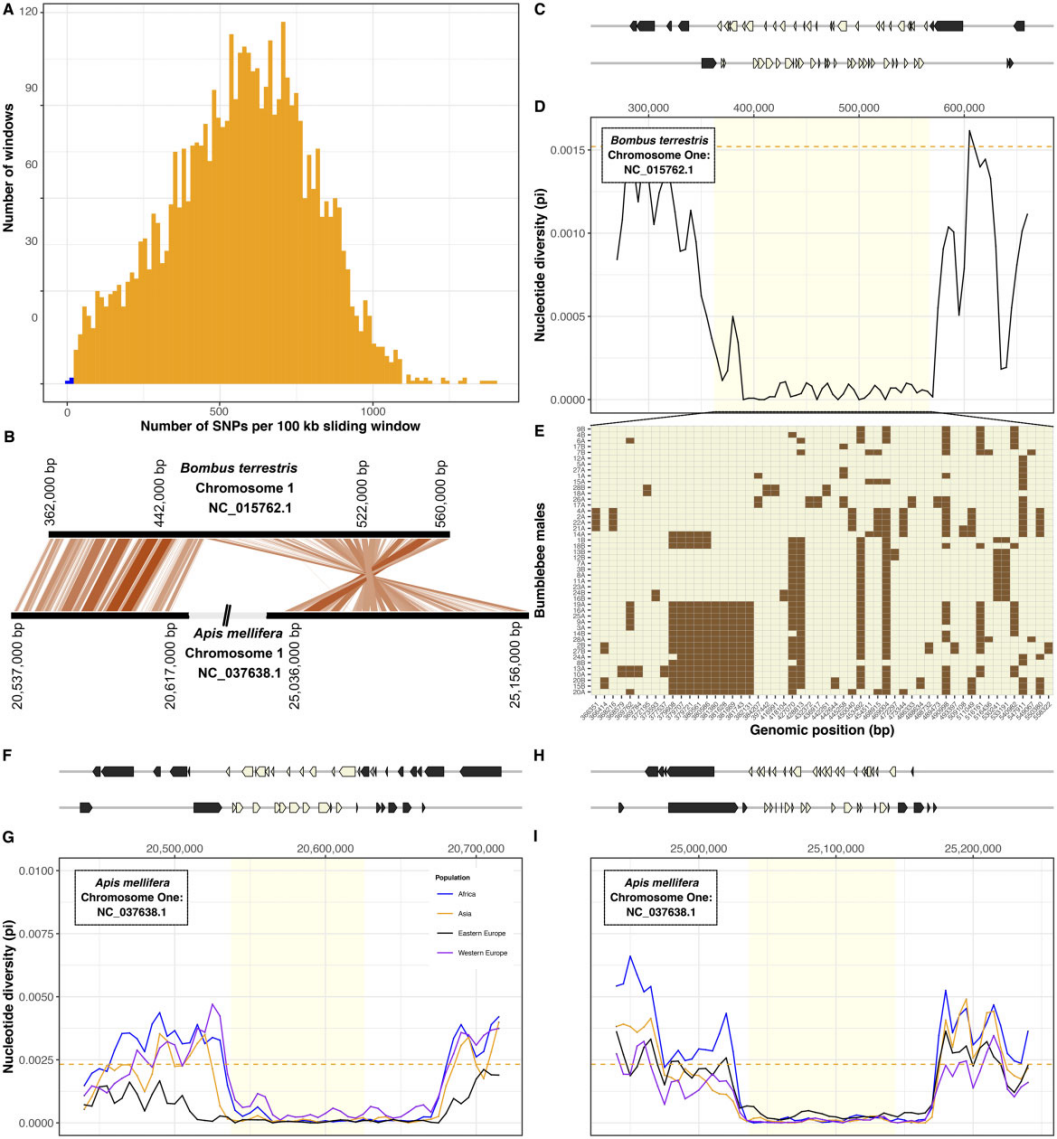
Conserved gene-rich region of low nucleotide diversity in bumblebee and honeybee. (*A*) Number of SNPs identified in 100 kb sliding windows across the bumblebee genome. The two windows with the lowest number of SNPs (in blue) are adjacent to each other on chromosome one. (*B*) Relative genomic positions of homologous regions of low diversity on chromosomes one of *Bombus terrestris* and *Apis mellifera*. (*C*) Genomic coordinates of 53 genes present in (beige) and flanking (gray) the region of low diversity. (*D*) Nucleotide diversity (*π*, calculated in 10 kb sliding windows) is low in this region in comparison with flanking regions and to the genome-wide mean (dashed line). (*E*) Genotypes for each of 46 *B. terrestris* males (rows) at each SNP (columns; chromosomal coordinate shown in the *x*-axis). Colors indicate reference allele (beige) or alternative allele (brown). (*F***–***I*) In the honeybee *Apis mellifera*, homology with the region of low diversity in *B. terrestris* is split between two regions. For both regions, we show genomic positions of genes (*F*, *H*). In four populations, both regions have lower nucleotide diversity (*π*, calculated in 10 kb sliding windows) than flanking regions or the rest of the genome (dashed line; *G*, *I*).

A 200,000 nucleotide-long region stood out in our analysis because it contains only 56 SNPs and thus has 21-fold lower nucleotide diversity (*π*∼7 × 10^−5^) than the genome-wide average (*π* = 1.5 × 10^−3^; *t*_df=46_ = 90.4, *P *<* *10^−15^; fig. 3). Furthermore, the low-diversity region has particularly high gene density (53 genes; *z*-score<-2σ) and represents a solid haplotype, with a population-derived recombination rate 298× lower (*ρ* = 1.2×10^−4^) than the genome-wide average (*ρ* = 0.035, *t*_df=1__,__226__,__700_ = −460, *P *<* *10^−15^).

To test whether the characteristics of this region are specific to *B. terrestris*, we identified orthologous regions in another bumblebee species *Bombus impatiens* and in the honeybee *Apis mellifera*. The orthologous region in *B. impatiens* contains 52 of the 53 genes and similarly has lower diversity (*π*∼1.7 × 10^−4^) than other regions (genome-wide average *π* = 1.2 × 10^−3^; *t*_df=18_ = −22.8, *P *<* *10^−15^). Orthology to the honeybee is split between two regions separated by 4.4 Mb, indicating that rearrangements have occurred because the common ancestor of bumblebees and honeybees existed at least 78 Ma. For both regions, honeybee populations had at least 13-fold lower nucleotide diversity than the rest of the genome (region 1: *t*_df=19.328_ = −98.9, *P *<* *10^−15^; region 2: *t*_df=15.8_ = −65.2, *P *<* *10^−15^; fig. 3). These patterns indicate that an intrinsic long-standing process is responsible for the low genetic diversity of these regions in bees. Although the regions include genes that are likely under strong purifying selection (supplementary table S7, Supplementary Material online), no particular gene annotation was overrepresented which could help interpretation. Unlike the rest of the genome, the lack of genetic diversity in this large region suggests that bees will have limited ability to adapt to selection pressures involving the genes it contains.

#### Selection on Potential Insecticide Susceptibility Genes

Selection for resistance to neurotoxic pesticides can lead to changes in expression or sequence of target receptors or detoxification enzymes in insect pests (Ffrench-Constant 2013). Because bumblebees can be exposed to pesticides when foraging on crops, and given the extensively documented detrimental effects of pesticide exposure on bumblebee health (Vanbergen 2013; Goulson et al. 2015), we preliminarily examined signatures of recent selection in genes for which orthologs in other species are known to be involved in responses to insecticide exposure. Four target receptors of insecticides were among the 10% of genes with the highest |*nS*_L_| scores: three nicotinic acetylcholine receptor subunits which are targets of neonicotinoid insecticides (*nAChR1a*, *nAChR6a*, *nAChR7a*; |*nS*_L_|>3.12 for all; supplementary table S8, Supplementary Material online), and *metabotropic glutamate receptor 2* (|*nS*_L_|=3.59), a target of the natural plant toxin l-canavanine (Mitri et al. 2009). Mutations in orthologs of two of the nicotinic acetylcholine receptor subunit genes confer resistance to neonicotinoid exposure in other species (Puinean et al. 2013; Shimada et al. 2020). Among genes putatively involved in detoxification, five cytochrome P450s and four carboxylesterases had strong signatures of selection (top 10% of genes with high |*nS*_L_| scores [|*nS*_L_|>2.89]; supplementary table S8 and fig. S7, Supplementary Material online). We also found strong signatures of selection for 42 genes that are differentially expressed in bumblebees after exposure to neonicotinoid pesticides (Bebane et al. 2019; Colgan et al. 2019; supplementary table S9, Supplementary Material online). There was no overlap between these 42 genes and those previously identified as having a role in insecticide resistance. Future research will help pinpoint the reasons for the patterns we observe, and whether some of the recent changes may reduce susceptibility to toxins naturally present in pollen and nectar, or to synthetic pesticides.

## Discussion

Human-induced environmental changes add to long-standing ecological and evolutionary challenges faced by wild animals. Identifying potential causes of pollinator declines has to date relied on inferences from laboratory experiments or on correlative associations in the field. Our study takes an important step toward understanding the bases of resilience of an important pollinator species by uncovering signatures left in the organism’s genetic “blueprint” in response to selective pressures. The strong signatures of selection we find at loci distributed throughout the *B. terrestris* genome are consistent with the view that insect pollinators face many different pressures. Environmental pressures likely contributed to recent changes that occurred in *B. terrestris* genes underlying physiology, neurology, and wing development.

Bumblebees also face intra- and interspecies pressures including, competition for food, habitat, and mates, and pressures from predators, pathogens, and parasites. Large-scale gene expression and functional genomic data sets are only beginning to be produced for bumblebees (López-Osorio and Wurm 2020) and will be crucial for disentangling how the specific changes we observed affect phenotypes and fitness. Similarly, historical sampling of museum specimens could help characterize changes over time in morphology, population structure, and allele frequencies.

The fine tuning of adaptive responses in *B. terrestris* is highlighted by our finding of strong signatures of selective sweeps within few nucleotides of neutrally evolving loci. Several characteristics of this species likely facilitate this fine tuning. Crucially, the high recombination rate (Liu et al. 2017) and social lifestyle of *B. terrestris* mean that one queen can produce hundreds of haploid males, encompassing a broad diversity of allelic combinations. These males are fully exposed to the environment as they spend weeks foraging and trying to attract a mate (Wolf et al. 2012). Male bees are also subject to haploid selection, which should lead to faster adaptation than in diploid species (Meisel and Connallon 2013; Pracana et al. 2021). Furthermore, the broad gene flow and large population size of *B. terrestris* enables the maintenance of large amounts of genetic diversity and the rapid spread of adaptive alleles. Although our data would be unable to detect slight changes in population size over the past century, our data and analyses support the absence of major recent population bottlenecks in this species. Future comparisons with sister species including those that are declining will clarify whether *B. terrestris* may have additionally harbored a generalist genetic toolkit further predisposing it to resilience.

We show that locating recent signatures of selection throughout the genome can indicate which genes and pathways changed for *B. terrestris* to adapt in response to the pressures it has faced. Furthermore, the amount and distribution of genetic diversity we observed throughout its genome suggest that this bumblebee species maintains an ability to respond to future pressures. Our work in this bumblebee species complements recent efforts in vertebrates and model systems. Future comparative genomic studies with other social and solitary pollinators will improve our ability to disentangle why species differ in their resilience to recent environmental changes. Additionally, scaling up our approach will enable the creation of frameworks for predicting detailed responses to environmental challenges for entire ecological networks. Overall, although insect declines are worrying, we show how at least one common pollinator is adapting.

## Materials and Methods

### Bumblebee Collection, DNA Extraction, and Sequencing

In the summer of 2014, we collected up to two males from each of 28 sites, with each site being >20 km from the nearest neighboring site (fig. 1*B*). Male *B.terrestris* (large earth or buff-tailed bumblebee) were caught using butterfly nets and transferred into individual 100-ml pots after morphological confirmation of sex and species. Pots were placed into a bag at 4–10 °C. Within 2 h, males were rapidly transferred to 2 ml cryotubes and then snap frozen in liquid nitrogen. Subsequent storage was at −80 °C.

From each bee, dissected tissue was homogenized in 200 μl of phenol in a 2-ml screw-cap tube (supplementary table S1, Supplementary Material online). Subsequently, DNA was extracted using phenol-chloroform followed by purification with the Sigma GenElute Mammalian Genomic DNA miniprep kit. DNA purity was initially assessed using a NanoDrop spectrophotometer (Thermo Fisher Scientific, United Kingdom) followed by quantification with a Qubit v3 fluorometer (Thermo Fisher Scientific). DNA from each male was fragmented to ∼550 bp using a Covaris M220 ultrasonicator and fragment size distribution assessed using a TapeStation 2200 (Agilent Technologies, United Kingdom). From each sample, we prepared an individually indexed Illumina TruSeq PCR-free DNA library, which was quantified using qPCR MasterMix (ABI Prism) and primer premix (Kapa Biosystems, United Kingdom). Libraries were pooled in equimolar concentrations and pairs of 125-bp sequences were produced on two lanes of Illumina HiSeq 2500 at Biomedical Research Centre Genomics, London, United Kingdom. Five samples were additionally sequenced on one lane of Illumina HiSeq 2500 at Oxford Genomics, Oxford, United Kingdom.

### Quality Assessment and Filtering of Raw Illumina Sequences

We obtained 616 million paired-end reads from the 51 samples we initially collected. Using bowtie2 (v.2.2.5; Langmead and Salzberg 2012) with the parameter “-X 1000,” we aligned raw reads to the *B. terrestris* reference genome (GCF_000214255.1; Sadd et al. 2015). The 422× cumulative genome coverage provided strong power to detect sites with nucleotide sequence polymorphism. Four males were removed from all biological analyses due to low coverage. A fifth male was only used for the analysis of contaminant sequences (supplementary appendix, Supplementary Material online) because >58.1% of reads from this male lacked similarity to the reference genome. The mean mapped coverage for each of the remaining 46 samples was 11.8× (min: 7×; max: 26.7×). Quality of raw reads was assessed using FastQC (v.0.11.3; https://www.bioinformatics.babraham.ac.uk/projects/fastqc; last accessed June 30, 2015). Illumina adapters were detected and removed using Trimmomatic (v.0.33; Bolger et al. 2014). Using Khmer (v.2.1.1; Crusoe et al. 2015) to first interleave pairs of reads, we removed sequences of low quality (where >25% of the read has a Phred quality score of strictly <20) using the fastx toolkit (v.0.0.14; http://hannonlab.cshl.edu/fastx_toolkit; last accessed November 30, 2015). We used Khmer to remove 31-mers present three or fewer times across the entire data set, as they likely represent technical artifacts or particularly rare variants that we would be unable to analyze. Sequences shorter than 50 bp were removed using seqtk (v.1.0-r82-dirty; https://github.com/lh3/seqtk; last accessed November 30, 2015). The final cleaned data set thus comprised 46 males with a mean coverage of 11.1× (min 6.7×; max 24.4×). This cleaned data set provides sufficient power to genotype the majority of polymorphic sites because 96.2% of the genome had >1× coverage in each of the 46 males. Overall, ∼99% of the reference genome had at least 20× coverage.

### Identification of Polymorphic Sites and Genotyping of Individuals

After mapping cleaned reads to the reference assembly using bowtie2, we called variants using freebayes (v.1.0.2-29-g41c1313; Garrison and Marth 2012) with the following parameters: –report-genotype-likelihood-max –use-mapping quality –genotype-qualities –use-best-n-alleles 4 –haplotype-length 0 –min-base-quality 3 –min-mapping-quality 1 –min-alternate-fraction 0.25 –min-coverage 1 –use-reference allele. We first removed the aforementioned five low-coverage individuals as they were each missing >10% of genotype calls, thus retaining data from 46 males. We then removed entire SNPs with low genotype quality scores (–minQ 20) and variants in collapsed repetitive regions (–max-mean-DP 100) using VCFtools (v.0.1.15; Danecek et al. 2011). We removed sites that appeared to be heterozygous, which is impossible in haploids, and all sites with more than two alleles as they also likely represent collapsed regions in the reference genome. To further reduce data set complexity, we used –remove-indels to only consider SNPs. We calculated allele frequencies and retained genotypes only where the rare allele was present in at least two males. Finally, we only considered those SNPs in regions of the genome that are mapped to the 18 linkage groups (representative of chromosomes). Mean nucleotide diversity π was calculated using 10 kb sliding windows with 5 kb overlap using PopGenome (v.2.2.4; Pfeifer et al. 2014).

### Assessment Population Structure

We investigated potential relatedness among collected bumblebees by performing IBS analyses on a pruned set of SNPs generated by SNPRelate (v.1.8.0; Zheng et al. 2012) using parameters that are similar to those previously used for *Drosophila* (Schmidt et al. 2017) (–ld-threshold = 0.2 –slide.max.n = 500). We further investigated population structure using three approaches with unpruned SNPs: principal component analysis using SNPRelate, ADMIXTURE (v.1.3.0; Alexander et al. 2009) with *K = *1–40 using cross-validation (–cv) as a measure to identify the best *K* value, and the linkage-aware approach fineSTRUCTURE (v.0.1.0; Lawson et al. 2012).

### Evidence of Recent Selective Sweeps

First, we identified regions of the genome with particularly low nucleotide diversity, indicative of “hard” sweeps. Second, to identify potential “soft” selective sweeps, we calculated *nS*_L_ (Ferrer-Admetlla et al. 2014) for all high confidence SNPs using selscan (v.1.1.0b; Szpiech and Hernandez 2014). This metric is a measure of extended haplotype homozygosity. We normalized all *nS*_L_ scores against the empirical genome-wide distribution using selscan “norm,” using default settings. We used the top 1% (|*nS*_L_| ≥ 2.56) absolute score of the *nS*_L_ metric (|*nS*_L_|) for all downstream analyses because |*nS*_L_| ≥ 2 indicates a selective sweep. Normalized |*nS*_L_| scores per gene, as well as NCBI RefSeq gene symbol and description, are provided in supplementary table S2, Supplementary Material online.

## Code Availability

The scripts underpinning our analysis are available from: https://github.com/Joscolgan/bter_population_genomics; last accessed January 1, 2021 and at https://wurmlab.com/data; last accessed January 1, 2021.

## Supplementary Material

Supplementary data are available at *Molecular Biology and Evolution* online.

## Supplementary Material

msab366_Supplementary_Data
